# Successful extreme foot revascularization with plaque cracking (percutaneous direct needle puncture of calcified plaque) technique of medial tarsal artery

**DOI:** 10.1016/j.jvscit.2023.101282

**Published:** 2023-08-01

**Authors:** Nicola Troisi, Francesco Canovaro, Daniele Adami, Raffaella Berchiolli

**Affiliations:** Vascular Surgery Unit, Department of Translational Research and New Technologies in Medicine and Surgery, University of Pisa, Pisa, Italy

**Keywords:** Below-the-ankle, PIERCE technique, Retrograde access, Tarsal artery

## Abstract

The percutaneous direct needle puncture of calcified plaque technique is a valuable method to allow for extreme revascularization of occluded below-the-ankle vessels. We report the case of an antegrade recanalization technique from the peroneal artery to medial plantar artery to achieve external “cracking” of a calcified plaque of the medial tarsal artery.

Chronic total occlusions of the infrapopliteal vessels can be difficult to recanalize. Below-the-knee and below-the-ankle (BTA) arteries are often highly calcified.^1^ Some cases of retrograde puncture of below-the-knee and BTA vessels to “crack” the atherosclerotic plaque with insertion of the needle inside the arterial lumen (PIERCE [percutaneous direct needle puncture of calcified plaque] technique) have been reported. Some of these cases related to BTA vessels have been reported as the “inner PIERCE technique.”^2^^,^^3^

We report the case of antegrade recanalization from the peroneal artery to medial plantar artery using a modified PIERCE technique with a radial cannulation needle used to crack the calcified plaque of the medial tarsal artery. The patient provided written informed consent for the report of his case details and imaging studies.

## Case report

An 82-year-old man was admitted to our outpatient service with chronic limb-threatening ischemia (Rutherford class 5; WIfI [wound, ischemia, and foot infection] classification score 1 [wound], 2 [ischemia], 0 [foot infection]). The preoperative toe brachial index was 0.2. Duplex ultrasound detected high-grade stenosis of the femoral bifurcation involving the ostia of the superficial femoral artery and profunda femoris, patency of the superficial femoral and popliteal arteries, and occlusion of the anterior tibial and posterior arteries, with a patent peroneal artery (GLASS [Global Anatomic Staging System] II). The patient underwent hybrid revascularization.^4^

A femoral endarterectomy and patch closure with bovine pericardium (VascuGuard; Baxter Healthcare) was performed. Next, an antegrade 5F sheath was inserted. Preoperative angiography confirmed occlusion of the anterior tibial artery and posterior tibial artery, with patency of the peroneal artery. The outflow of the peroneal artery included some calcaneal branches (Supplementary Video 1, online only).

**Fig 1:**
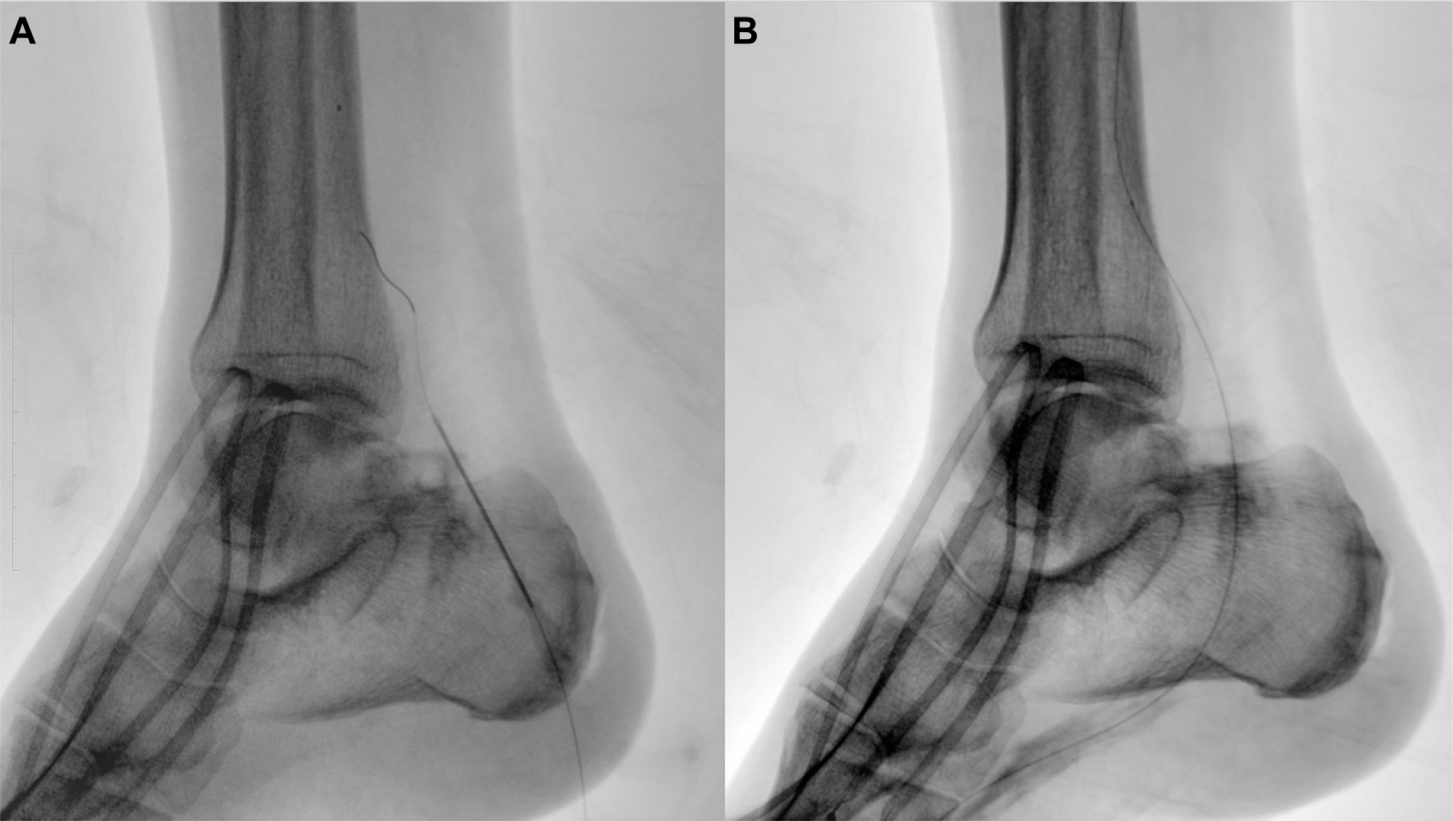
**a,** Percutaneous direct needle puncture of calcified plaque (PIERCE) technique in the medial tarsal artery. **b,** Antegrade recanalization of the medial plantar artery.

**Fig 2:**
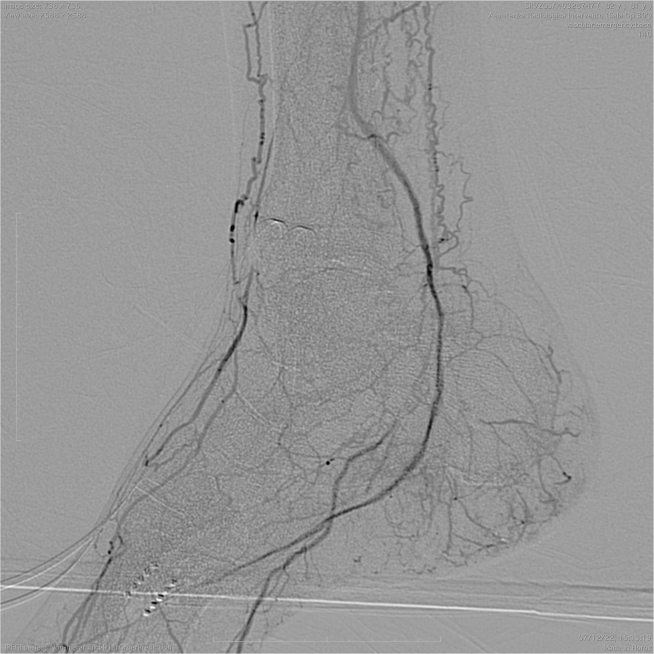
Completion angiography.

Antegrade recanalization of the anterior and posterior tibial arteries was unsuccessful. Under fluoroscopic guidance, high-grade calcifications of the medial tarsal artery were detected (connection between the peroneal artery and medial plantar arteries). An antegrade approach with different 0.014-in. guidewires (Advantage; Terumo Medical Corp; and Command ES; Abbott Vascular) was unsuccessful to recanalize the medial tarsal artery owing to the presence of high-grade calcifications. Thus, under ultrasound guidance, direct retrograde puncture of the highly calcified medial tarsal artery was performed with a 20-guage radial cannulation needle. Five shots in total were performed. This allowed for progression of a 0.014-in. Advantage guidewire (Terumo Interventional Systems) in a retrograde fashion to reach the true lumen into the peroneal artery (Fig 1, *a*). After plaque cracking and retrograde recanalization, it was possible to remove the guidewire from below and to reach from above the true lumen of the medial tarsal artery with a 0.014-in. Advantage guidewire to connect the peroneal artery to the medial plantar artery (Fig 1, *b;* Supplementary Video 2, online only). Next, standard balloon angioplasty (Armada XT, 1.5 × 20 mm; Abbott Vascular; and Passeo-14, 2 × 100 mm and 2.5 × 220 mm; Biotronik SE & Co) was performed. Completion angiography detected a direct flow from the peroneal artery to the foot with successful direct angiosome revascularization (Fig 2). Intraoperatively, continuous monitoring of transcutaneous oximetry was performed on the foot, which was +7% after revascularization.

**Fig 3:**
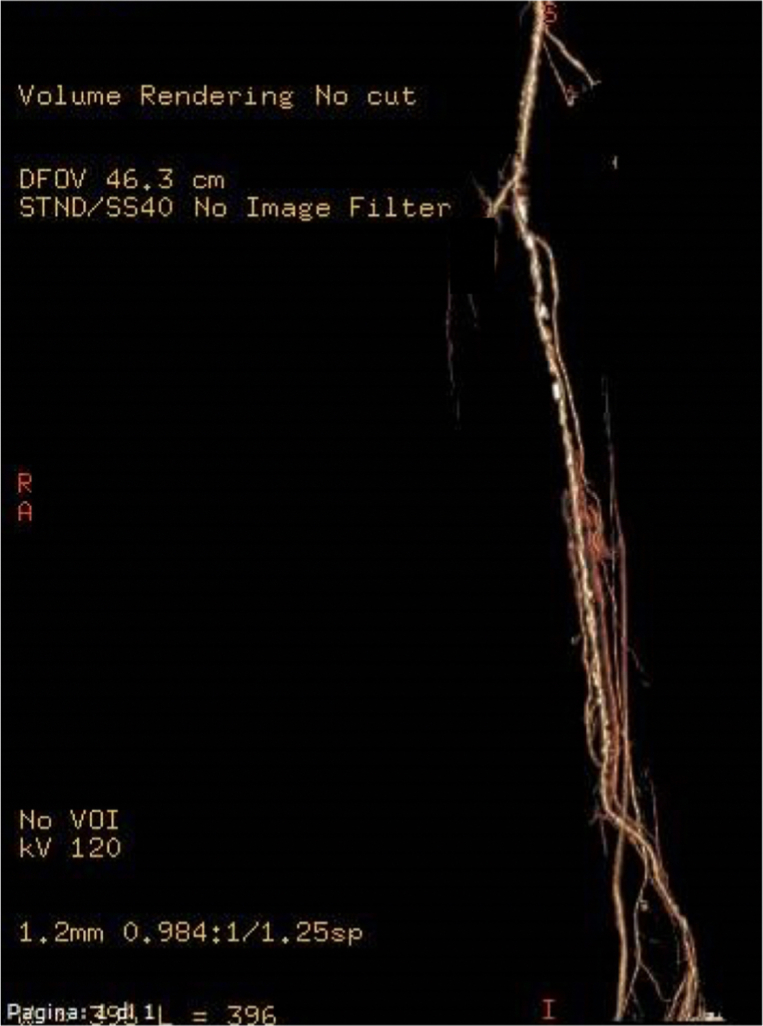
Computed tomography scan at 3 months of follow-up.

The patient was discharged on postoperative day 10 with dual antiplatelet therapy (aspirin and clopidogrel). In the immediate postoperative period, the patient developed fever and increased white blood cell count. Because of the high suspicion of osteomyelitis, a computed tomography scan was performed (Fig 3). At 3 months, the patient had no rest pain, and the calcaneal lesion was almost healed. Duplex ultrasound detected patency of the peroneal and medial plantar arteries with acceptable flow (peak systolic velocity, 71 cm/s). The toe brachial index for the first toe was 0.92.

## Discussion

For patients with chronic limb-threatening ischemia, revascularization with a direct groin-to-foot target artery pathway is the gold standard for treatment.^4^ When an endovascular approach is used, antegrade or retrograde recanalization will sometimes not successfully guarantee direct flow to the foot.^5^ In the literature, extreme retrograde approaches have been proposed with direct puncture of foot arteries.^6^ However, plaque cracking of tarsal vessels has not yet been reported. The technique we describe in the present report is a modified PIERCE technique performed in association with the SAFARI (subintimal arterial flossing with antegrade–retrograde intervention) technique.^2^^,^^5^ It can be used in patients with extremely calcified BTA vessels, where it is not possible to obtain antegrade recanalization.^7^ In experienced hands, plaque cracking can be performed under ultrasound guidance. The main risk of this technique is to “burn” the reentry sites. Thus, we recommend cracking the plaque in the mid-portion of the calcification and then performing vessel recanalization to ensure reentry into the true lumen. Therefore, we decided to use a 20-gauge cannula for radial cannulation to have less aggressive plaque cracking. In addition, balloon angioplasty should be performed after reaching the true lumen from above and removal of all the devices used from the retrograde passage. Intravascular lithotripsy could be an alternative option to better manage the calcium burden.

Some contraindications to this technique should be considered, including an extensive wound that would not allow the “external” vessel puncture and the lack of strict connections between the two vessels to be connected to guarantee a target artery pathway. For our patient, a duplex ultrasound scan was very useful to evaluate the presence of calcifications in the communicating plantar vessels.

## Conclusions

The cases reported demonstrate the feasibility of a modified PIERCE technique with plaque cracking of the medial tarsal artery to obtain a connection between the peroneal and medial plantar arteries. The risk of “burning” the reentry sites is very high; thus, this technique should be considered for very select patients when the antegrade recanalization is not feasible after many attempts.

## Appendix (online only)

Supplementary Video 1 (online only) Preoperative angiography showing unsuccessful attempt to obtain standard antegrade recanalization.

Supplementary Video 2 (online only) Angiography showing antegrade recanalization with direct flow into the medial plantar artery.

## References

[1] Tan M., Ueshima D., Urasawa K. (2021). Prediction of successful guidewire crossing of below-the-knee chronic total occlusions using a Japanese scoring system. J Vasc Surg.

[2] Nakama T., Muriashi M., Obunai K., Watanabe H. (2020). Efficacy of the novel inner PIERCE technique for severely calcified below-the-knee occlusions in a patient with chronic limb-threatening ischemia. Catheter Cardiovasc Interv.

[3] Takei T., Miyamoto A., Takagi T., Yamamuchi Y. (2021). A novel technique of percutaneous intraluminal cracking using a puncture needle for severe calcified lesions of below-the-knee and below-the-ankle arteries. Diagn Interv Radiol.

[4] Conte M.S., Bradbury A.W., Kolh P. (2019). Global vascular guidelines on the management of chronic limb-threatening ischemia. J Vasc Surg.

[5] Huillca M.A., Moreno-Loaiza M., Tipacti-Rodríguez F., Briceño-Alvarado M., Llalle W.S.C. (2021). Endovascular revascularization of chronic total arterial occlusion of the lower limb using the SAFARI technique. J Vasc Bras.

[6] Palena L.M., Manzi M. (2012). Extreme below-the-knee interventions: retrograde transmetatarsal or transplantar arch access for foot salvage in challenging cases of critical limb ischemia. J Endovasc Ther.

[7] Solimeno G., Salcuni M., Capparelli G., Valitutti P. (2022). Technical perspectives in the management of complex infrainguinal arterial chronic total occlusions. J Vasc Surg.

